# Pixel-wise navigation line extraction of cross-growth-stage seedlings in complex sugarcane fields and extension to corn and rice

**DOI:** 10.3389/fpls.2024.1499896

**Published:** 2025-01-30

**Authors:** Hongwei Li, Xindong Lai, Yongmei Mo, Deqiang He, Tao Wu

**Affiliations:** ^1^ School of Mechanical Engineering, Guangxi University, Nanning, China; ^2^ College of Engineering, South China Agricultural University, Guangzhou, China

**Keywords:** classical image processing, crop seedling, navigation line extraction, plantation row, growth stage

## Abstract

Extracting the navigation line of crop seedlings is significant for achieving autonomous visual navigation of smart agricultural machinery. Nevertheless, in field management of crop seedlings, numerous available studies involving navigation line extraction mainly focused on specific growth stages of specific crop seedlings so far, lacking a generalizable algorithm for addressing challenges under complex cross-growth-stage seedling conditions. In response to such challenges, we proposed a generalizable navigation line extraction algorithm using classical image processing technologies. First, image preprocessing is performed to enhance the image quality and extract distinct crop regions. Redundant pixels can be eliminated by opening operation and eight-connected component filtering. Then, optimal region detection is applied to identify the fitting area. The optimal pixels of plantation rows are selected by cluster-centerline distance comparison and sigmoid thresholding. Ultimately, the navigation line is extracted by linear fitting, representing the autonomous vehicle’s optimal path. An assessment was conducted on a sugarcane dataset. Meanwhile, the generalization capacity of the proposed algorithm has been further verified on corn and rice datasets. Experimental results showed that for seedlings at different growth stages and diverse field environments, the mean error angle (MEA) ranges from 0.844° to 2.96°, the root mean square error (RMSE) ranges from 1.249° to 4.65°, and the mean relative error (MRE) ranges from 1.008% to 3.47%. The proposed algorithm exhibits high accuracy, robustness, and generalization. This study breaks through the shortcomings of traditional visual navigation line extraction, offering a theoretical foundation for classical image-processing-based visual navigation.

## 1 Introduction

Sugarcane (*Saccharum officinarum*) is a vital sugar-producing and energy-producing crop widely cultivated in tropical and subtropical countries or regions (Wang W. et al., 2023), with an annual global yield of approximately 1.9 billion tonnes (FAO, 2022). Diverse field management tasks at the seedling stage, such as stumping, weeding, spraying pesticides, and root-zone soil backfill, are crucial in improving the sugarcane yield and quality. At present, these tasks severely rely on manual operation. In the face of the aging labor population and the increase in labor costs, it is urgently needed to explore intelligent agricultural machinery for field management. Intelligent agricultural machinery can be a potential resolution to perform field management tasks, and navigation is one of the key technologies to achieve intelligent detection/control of agricultural machinery.

Commonly, agricultural machinery navigation methods mainly include Global Navigation Satellite System (GNSS) navigation (Luo et al., 2009; Liu et al., 2018; Zhang et al., 2022), radar navigation (Blok et al., 2019), ultrasonic navigation (Chen et al., 2018) and visual navigation (Liu et al., 2024; Bai et al., 2023; Shi et al., 2023). According to the available literature, visual navigation attracted more interest in these methods. This phenomenon might occur because visual navigation, relying on the interpretation and judgment of various landscapes, can be effective in diverse settings. This includes extensive, continuous farmlands on plains and fragmented, small-scale farmlands in hilly and mountainous terrains.

It is well known that the visual navigation of crop seedlings can be classified into two categories: (I) the navigation based on the ridge centerline and (II) the navigation based on crop plantation-row centerlines. For the first category, in the early years, one typically representative method is the least-square-based navigation line detection algorithm of tomato ridge (Wang et al., 2012). In recent years, further advances have been found in this regard. For instance, Yang et al. (2020) proposed a method of real-time extraction of the navigation line between the corn rows based on a dynamic Region of Interest. Facing the higher corn seedlings, Gong et al. (2020, 2022) gave two corn field navigation line extraction methods by combining edge detection and area localization and integrating gradient descent and corner detection. Chen et al. (2020) investigated a median point Hough transformation algorithm to fit the navigation paths of greenhouse tomato-cucumber seedlings. Following this previous study, Chen et al. (2021) also investigated a prediction-point Hough transform to extract the navigation path for greenhouse cucumber seedlings. With the in-depth application of deep learning algorithms, ever-increasing researchers have adopted deep learning algorithms based on semantic segmentation technology to obtain navigation lines between the crop rows. For example, Rao et al. (2021) combined the U-net and Fast-Unet models to generate the navigation line. Zhao et al. (2021) used remote sensing images acquired from unmanned aerial vehicles and extracted corn field ridge centerline based on a fully convolutional network. Li et al. (2022a) achieved the navigation line extraction using a Faster-U-net model and adaptive multi-ROIs. Song et al. (2022) proposed a navigation algorithm based on semantic segmentation in wheat fields using an RGB-D camera. Yang et al. (2023) develops a real-time crop row detection algorithm for corn fields, leveraging YOLO neural network and ROI extraction to achieve high accuracy and robustness. For such a category, it is evident that navigation utilizing the ridge centerline depends on feature point extraction between the adjacent crop rows and the color difference in ridge and plantation rows. The limited condition of non-intersecting overlapping leaves between adjacent crop rows compounds this reliance on either classical image processing or deep learning algorithms. Especially for the semantic segmentation-based ridge centerline extraction, from the point of view of real-world application, it remains challenging to integrate with downstream low-cost, lightweight edge devices after obtaining semantic segmentation results.

As for the second category, navigation based on crop plantation-row centerlines has been a research hotspot in the past two decades. Most of the available literature in plantation-row centerlines extraction were based on the development of classical image processing algorithms, such as Hough transform based on connected component labeling (Rao and Ji, 2007), improved Hough transformation (Zhao et al., 2009; Diao et al., 2015; Wang et al., 2020), median-point Hough transform (Li et al., 2022b), least square method (Si et al., 2010; Ma et al., 2022), color model and nearest neighbor clustering algorithm (Zhang et al., 2012), scan-filter algorithm (Li et al., 2013), constraint of liner correlation coefficient (Meng et al., 2013), improved genetic algorithm (Meng et al., 2014), boundary detection and scan-filter (He et al., 2014), multi-ROIs (Jiang et al., 2015; Yang et al., 2020; Yu et al., 2021; Wang A. et al., 2021; Li et al., 2021), SUSAN corner (Zhang et al., 2015), Harris corner points (Zhai et al., 2016a), Census transformation (Zhai et al., 2016b), particle swarm optimization (Meng et al., 2016), SUSAN corner and improved sequential clustering algorithm (Zhang et al., 2017), image characteristic point and particle swarm optimization-clustering algorithm (Jiang et al., 2017), accumulation threshold of Hough transformation (Chen et al., 2019), sub-regional feature points clustering (Liao et al., 2019), Gaussian heatmap (He et al., 2022), contour computing with vertical projection (He et al., 2021), regional growth and mean shift clustering (Wang A. et al., 2021), binocular vision and adaptive Kalman filter (Zhai et al., 2022), RANSAC (Random Sample Consensus) algorithm (Li et al., 2022c), and region growth sequential clustering-RANSAC algorithm (Fu et al., 2023). These numerous literature revealed that both past and present, a significant proportion of researchers are still keen to perform navigation centerline extraction based on classical image processing algorithms. Classical image processing algorithms can be efficiently run on CPUs without the requirement for high-performance computing resources such as GPUs. When handling data from diverse sources and with varying characteristics, the compatibility and consistency of heterogeneous systems pose significant challenges. Classical image processing algorithms, due to their simplicity, are adaptable to various affordable systems and application scenarios, simplifying the complexities associated with managing heterogeneous data. Moreover, these algorithms are more explainable and easier to implement in low-performance computing platforms. Nevertheless, when facing complex image processing, some algorithm executions are time-consuming and can result in a limited generalization ability.

With the further popularity of deep learning technology, some researchers have attempted to develop various deep learning algorithms to extract navigation centerlines in the last few years to tackle this challenge. For instance, Lin et al. (2020) used the Faster R-CNN to detect the transplanting rice seeding in complex paddy field environments, obtaining mean absolute errors of a deviation of 8.5 mm in the lateral distance and 0.50° of travel angle. Zhang et al. (2020) extracted the centerlines of rice seedlings based on the YOLOv3 model, achieving a mean average precision of 91.47%, a mean average angle error of 0. 97° in the extracted centerline, and an average runtime of 82. 6 ms for one image. Diao et al. (2024) developed an algorithm for recognizing corn crop rows at different growth stages using the ST-YOLOv8s network. Their approach, which included a swin transformer-based backbone and a local–global detection method, demonstrated improved mean average precision (MAP) and accuracy compared to YOLOv5s, YOLOv7 and YOLOv8s, while reducing the average angle error and fitting time in crop row detection experiments. Li et al. (2023) presents E2CropDet, an efficient end-to-end deep learning model for crop row detection. Zheng and Wang (2023) used Pix2Pix Net to improve navigation line extraction ability in image pre-processing. Ruan et al. (2023) proposed a crop row detection method based on YOLO-R, attaining accuracy values of 93.91%, 95.87%, and 89.87% on the seven-day, 14-day, and 21-day for rice seedling row detection, respectively. Diao et al. (2023) developed a navigation line extraction algorithm based on an improved YOLOv8s network in corn seedlings, getting a significantly enhanced performance in mean average precision and F1, as compared to the YOLOv7 network and original YOLOv8 network. Liu et al. (2023) showed a recognition method of corn crop rows using the MS-ERFNet model, the mean intersection over union (mIoU) and the pixel accuracy (PA) of the MSERFNet model was 93.40% and 97.54%, respectively, which were higher than other models. Wang S. et al. (2021, 2023a, 2023b) adopted row vector grid classification, initial clustering, and exterior point elimination, as well as sub-region growth and outlier removal, to recognize straight or curved rice seedling rows, comprehensively realizing the centerline extraction of transplanted rice seedling rows under different paddy field environments.

Undoubtedly, deep learning algorithms in crop seedling centerline extraction require less execution time and showcase a better generalization ability. Despite these advantages, deep-learning-based methods require comprehensive datasets, considerable image labeling, innovative algorithm development, and rigorous parameter-adjusting processes. In addition, most of the deep learning algorithms lacked verifications in other crop seedlings; as a result, their generalization ability remains limited.

In conclusion, existing approaches often target specific crops, such as rice and corn, which typically exhibit uniformity due to agronomic influences, yet a generalizable navigation line extraction algorithm capable of adapting to various crops, growth stages, and environmental conditions remains absent. As a result, algorithms tailored to these crops may struggle with generalizability when applied to others, such as sugarcane, which has more complex and diverse characteristics. For instance, in sugarcane seedlings, ridge structure and spacing vary significantly, and little literature has explored this field. A noteworthy exception is Yang et al. (2022), who used LiDAR to extract navigation lines between sugarcane ridges. Moreover, traditional methods and many deep-learning-based approaches focus on site-specific, crop-specific, or growth-stage-specific ridge centerline navigation or plantation-row centerline navigation. A generalizable navigation line extraction algorithm that can handle cross-site, cross-crop, and cross-growth-stage scenarios has yet to be reported. Addressing this gap, our study emphasizes the development of an algorithm designed with broader applicability, aiming to bridge the limitations of the current literature.

In this study, a generalizable navigation line extraction algorithm based on classical image processing technologies was proposed. This study aims to extract the navigation line for cross-growth-stage sugarcane plantation rows under complex in-field environments using classical image processing technologies. The specific objectives of the study were to (a) propose an adaptive navigation line extraction algorithm of sugarcane plantation rows in different early growth stages, (b) validate the effectiveness of the proposed algorithm in complex sugarcane field environments, and (c) apply the proposed algorithm into the navigation line extraction of plantation rows in rice and corn, verifying its generalization capability.

## 2 Materials and methods

The proposed navigation line extraction method comprises four steps: image acquisition, image preprocessing, optimal region detection, and navigation line fitting.

### 2.1 Image acquisition

#### 2.1.1 Location 1 – sugarcane seedlings

The image acquisition location of sugarcane seedlings is situated in Guangxi subtropical new town of agri-forestry sciences, which belongs to Guangxi University, Guangxi Province, China (22.52°N, 107.79°E). The sugarcane seedling images were taken using a mobile device, Huawei Mate 50, positioned at a declination angle of about 45° relative to the terrestrial surface on May 2023. In total, 153 images were captured at different times.

#### 2.1.2 Location 2 – corn seedlings

Corn seedlings’ image acquisition location is in Guangxi University Experimental Base, Guangxi Province, China (22.86°N, 108.30°E). The corn seedling images were taken using the same Huawei Mate 50, positioned at a declination angle of about 45° relative to the terrestrial surface on August 2023. In total, 218 images were captured at different times.

#### 2.1.3 Location 3 – rice seedlings

The rice seedlings’ image acquisition location is in Guangxi University Experimental Base, Guangxi Province, China (22.86°N, 108.30°E). The rice seedling images were taken using the same Huawei Mate 50, positioned at a declination angle of 45° relative to the terrestrial surface on August 2023. In total, 135 images were captured at different times.

These acquired images cover diverse in-field conditions such as varying light, inter-row spacing variability, decay branches, overlapping leaves, soil color difference, and disordered vegetation, as well as multiple growth stages which are determined by the number of crop leaves such as ‘Code 12’ to ‘Code 19’ (Zadoks et al., 1974). To further expand the dataset and evaluate the generalization performance of the algorithm, this study employed the open-source image augmentation library Albumentations to enhance the dataset images. These augmentation operations simulated the distributions of sugarcane, corn, and rice seedlings under various low-light conditions and rainy scenarios.

In the following subsections, the sugarcane seedling images were first selected for algorithm development, while the other two image datasets were used for evaluating their generalization performance, as shown in sections 3.2 to 3.3.

### 2.2 Image preprocessing

**Figure 1:**
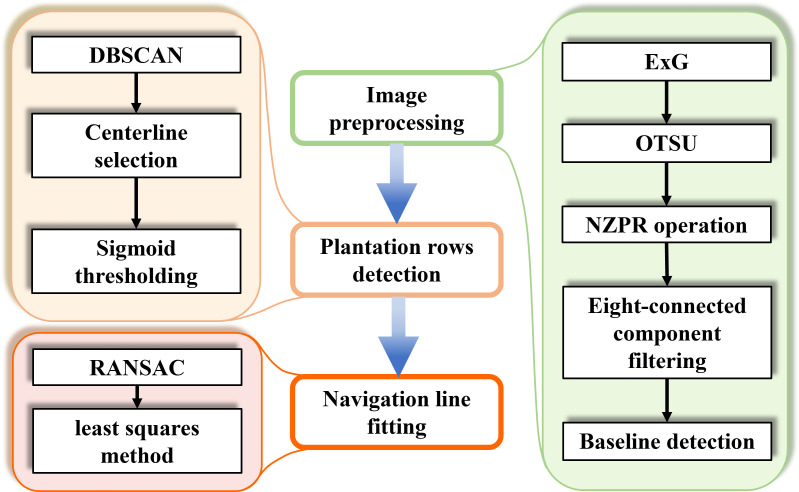
The overall architecture of image processing. Colored boxes indicate the detailed algorithms used for each key step.

The Windows 11 operating system was used to perform image processing tasks on a notebook with an AMD R7-7745HX and NVIDIA GeForce RTX 4060 with 8 GB VRAM. The algorithm in this paper was implemented in C++ using Microsoft Visual Studio 2022 as the integrated development environment. The flowchart of image processing is shown in **Figure 1**. These steps will be described further in the following sections.

#### 2.2.1 Grayscale transformation

To locate crop rows, the excess green index (ExG) is used to segment the vegetation and non-vegetation areas in the image based on the distinct difference in the green index (Woebbecke et al., 1995). The ExG is defined in Equation 1. Grayscale images can be obtained by applying the ExG, as shown in **Supplementary Figure S1B**.


(1) $$\begin{array}{c} Gray\left(x,y\right)=\{\begin{array}{cc} 0, & \;2ɡ-r-b<0\\ 255, & \;2ɡ-r-b>1\\ 255\left(2ɡ-r-b\right), & \;otherwise \end{array} \end{array}$$

where Gray(x,y)  denotes the gray level of the pixel at the position (x,y) and rgb are determined by the normalized values of the corresponding RGB channels respectively, as shown in Equation 2.


(2) $$\begin{array}{c} \{\begin{array}{c} r=\frac{R}{R\;+\;G\;+\;B}\\ ɡ=\frac{G}{R\;+\;G\;+\;B}\\ b=\frac{B}{R\;+\;G\;+\;B} \end{array} \end{array}$$

#### 2.2.2 OTSU thresholding and NZPR operation

Threshold segmentation using the Otsu method (Otsu, 1979) on grayscale images effectively extracts regions of interest. Let $p_{i}$ be the proportion of pixels for each grayscale level compared to the total number of pixels in the image, as shown in Equation 3. Let t be the threshold segmenting the image into foreground and background. Let $P_{1}$ and $P_{2}$ represent the probabilities of foreground and background, respectively. Let $m_{1}$ and $m_{2}$ represent the average grayscale values of the foreground and background, respectively. Let $m_{G}$ be the average grayscale values of the image. Thus, the variance between the two categories can be calculated by the Equations 3 and 4:


(3) $$\begin{array}{c} \;p_{i}=\frac{n_{i}}{N} \end{array}$$

(4) $$\begin{array}{c} \sigma_{B}^{2}=P_{1}{\left(m_{1}-m_{G}\right)}^{2}+P_{2}{\left(m_{2}-m_{G}\right)}^{2} \end{array}$$

where $n_{i}$ and N represent the sum of pixels for each grayscale level and total pixels of the image, respectively, while $P_{1}=\sum_{i=0}^{t}\;p_{i}$ , $P_{2}=\sum_{i=t+1}^{255}\;p_{i}$ , $m_{1}=\frac{1}{P_{1}}\sum_{i=0}^{\text{t}}\;ip_{i}$ , $m_{2}=\frac{1}{P_{2}}\sum_{i=t+1}^{255}\;ip_{i}$ and $m_{G}=\sum_{i=0}^{255}\;ip_{i}$ . The threshold $t^{*}$ that maximizes the inter-class variance can be calculated by the Equation 5:


(5) $$\begin{array}{c} t^{*}=argmax_{t}\sigma_{B}^{2}\left(t\right) \end{array}$$

where $t^{*}$ represents the optimal threshold.

**Figure 2:**
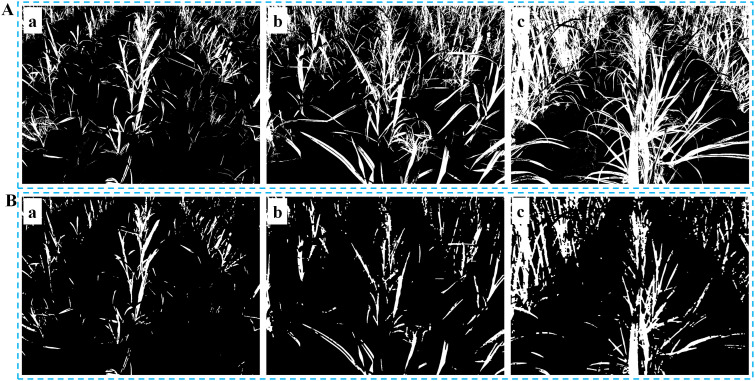
Threshold segmentation and morphological operation. **(A)** Binary images at different growth stages: (Aa) Slight occlusion, NZPR=11.84%; (Ab) Moderate occlusion, NZPR=22.32%; (Ac) Severe occlusion, NZPR=36.38%; **(B)** Images after morphological operation: the opening operation was applied in (Ba), (Bb) and (Bc) based on NZPR.


Once the threshold that corresponds to the maximum inter-class variance is defined, it becomes the optimal threshold for the segmentation. The binary image illustrated in **Figure 2A** can be obtained based on the following Equation 6:

(6) $$\begin{array}{c} Gray\left(x,y\right)=\{\begin{array}{ll} 255, & \;Gray\left(x,y\right)>ɡ\\ 0, & \;otherwise \end{array} \end{array}$$

where $g=kt^{*}$ , in which k is a proportion coefficient. According to pre-test, k=0.8 is an optimal proportion coefficient to extract the green features of sugarcane to the binary image.

Given that non-zero pixels in the binary image represent crops, this study proposed a concept of non-zero pixel ratio (NZPR), which denotes the proportion of non-zero pixels relative to the total number of pixels in the image to describe the different growth stages of crops, as Equation 7:


(7) $$\begin{array}{c} NZPR=\frac{\sum_{x,y}Gray\left(x,y\right)/255}{N}\times 100\% \end{array}$$

Furthermore, it is observed that the sugarcane seedlings exhibit strong branching ability of their leaves from the seedling stage (**Figure 2A**). As the growth stage advances, the branching density and coverage of the leaves increase continuously, resulting in severe occlusion of the leaf canopy of adjacent crop rows (**Figure 2A**c), which leads to unfavorable repercussions for subsequent clustering processes.

It is worth noting that different agricultural scenarios have varying requirements regarding image resolution. Low-resolution images, which do not require excessive image details, offer better real-time performance. On the other hand, high-resolution images provide more precise information for tasks such as planting and weed control. Given common resolutions used in typical cameras, the image acquired with a resolution of 4096×3072 is converted into two-type images with 640×480 and 1920×1080. As a result, established mathematical models were developed to correlate the two different resolutions—640×480 and 1920×1080—with the application of morphological operations.

To effectively tackle the severe occlusion of the leaf canopy of adjacent crop rows, a morphological operation (erosion followed by dilation) was implemented to eliminate extraneous branches and slight noise. According to pre-test, the iterations of erosion and dilation are determined by our defined NZPR ranges, as shown as below.

1. For images with a resolution of 640×480, its $n_{erosion}$ can be described as


(8) $$\begin{array}{c} n_{erosion}=\{\begin{array}{cc} 0, & NZPR<6\%\\ round\left(-4.0229\cdot NZPR^{2}+11.7543\cdot NZPR-0.17\right), & 6\%\leq NZPR\leq 50\text{\% }\\ 5, & NZPR>50\% \end{array} \end{array}$$

2. For images with a resolution of 1920×1080, its $n_{erosion}$ can be described as


(9) $$\begin{array}{c} n_{erosion}=\{\begin{array}{cc} 0, & NZPR<6\%\\ round\left(53.5714\times NZPR^{2}+3.9286\cdot NZPR+0.0714\right), & 6\%\leq NZPR\leq 20\%\\ 10\times NZPR+2, & 20\%<NZPR\leq 50\%\\ 8, & NZPR>50\% \end{array} \end{array}$$

(10) $$n_{dilation}=\lfloor \frac{n_{erosion}}{2}\rfloor$$

Compared to linear relationships, nonlinear relationships are more effective in balancing the preservation of image details with the suppression of unnecessary pixels. However, in cases where the NZPR is less than 6%, there are not enough feature pixels in the image, and therefore, erosion is not necessary.

As sugarcane seedlings grow, the NZPR value steadily increases (**Figure 2**), requiring more corrosion and expansion iterations, leading to more significant differences between them. As shown in **Figure 2B**, the main stems of sugarcane seedlings were retained in the binary image after the morphological operation. Specifically, a 3×3 kernel was used in this process.

#### 2.2.3 Connected component filtering and baseline detection

The morphological operations effectively suppressed extraneous branches and leaves in the binary image. However, it was also observed that numerous disconnected noises were introduced into the image, resulting from erosion and dilation. At the same time, the relatively larger connected components constitute the significant parts of the crop.

Based on the observed difference mentioned above, an eight-connected labeling algorithm that considers the pixel neighborhood in all eight directions was applied to identify individual objects. The specific steps for the labeling process are described as follows:

Step 1. Initialize a counter that indicates the count of currently discovered connected components. Initialize a label image to store the label of each pixel.
Step 2. Iterate through each pixel in the input image. Based on the grayscale value of the current pixel, classify it into foreground and background. The presence of a non-zero grayscale value denotes the membership of a pixel to the foreground and, in some cases, to a particular connected component. Identify the labelled pixels within its neighborhood and place them into a set.
Step 3. Identify the smallest element in the set, corresponding to the minimum label value, and assign it to a variable. This variable represents the label of the connected component to which the current pixel might belong. If the set is empty, it indicates that the current pixel has no labelled neighboring pixels, signifying it as the starting point of a newly discovered connected component. Therefore, increment the counter by one and assign this value as the label of the current pixel. If the set is not empty, assign the variable mentioned above as the label of the current pixel.
Step 4. Replace different labels belonging to the same connected component with a single label, referred to as merging equivalence class (MEC). MEC ensures that each connected component has a unique label.

The corresponding algorithm for the connected component labeling process is presented in **Algorithm 1**.

Algorithm 1 **The algorithm of the labeling process.**

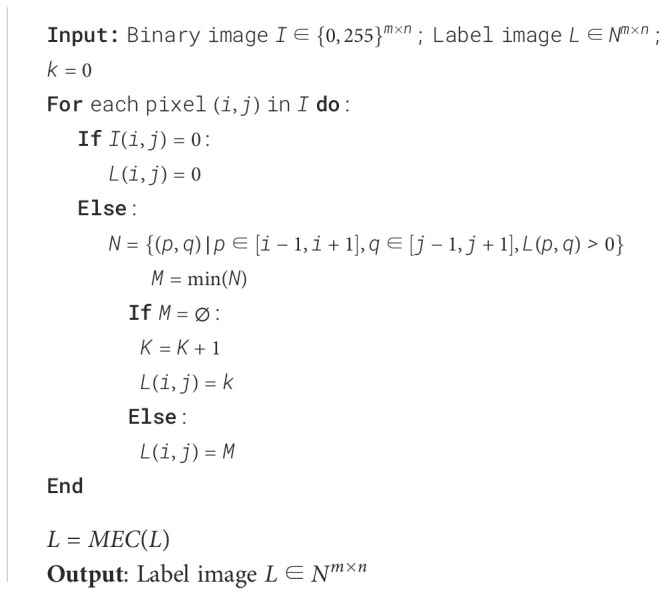

**Figure 3:**
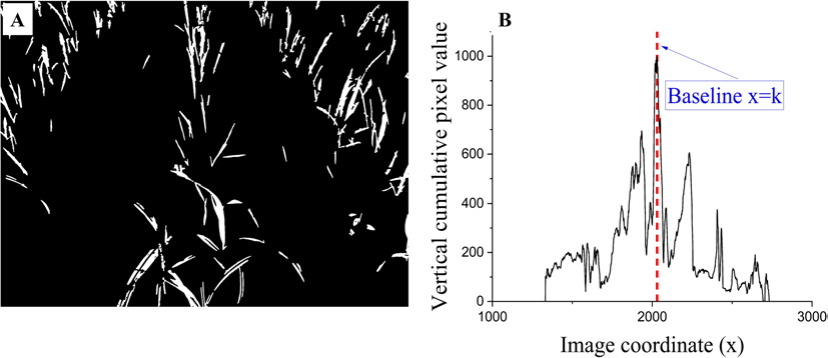
Connected component detection and vertical projection. **(A)** Eight-connected component filtering image; **(B)** Vertical projection curve from the filtering image, the value of (k) depends on the maximum column pixel value of each image.


Furthermore, a pixel re-binarization operation, such as in Equation 11, was employed to eliminate small connected components considered residual pixels after performing morphological opening. **Figure 3A** illustrates the image that was processed.

(11) $$\begin{array}{c} B\left(i,j\right)=\{\begin{array}{ll} 255, & \;L\left(i,j\right)>0\text{ \& }A\left(L\left(i,j\right)\right)\geq M\times C\\ 0, & \;otherwise \end{array} \end{array}$$

where A(L(i,j)) represents the area of the connected component to which the current pixel L(i,j) belongs, M represents the mean area of all connected components, C denotes a given coefficient, and pre-test has shown that a value of 0.7 achieves better elimination effects.

To extract the center row of sugarcane seedlings in the image, a baseline is needed to predict its potential position. The baseline detection is based on the following two assumptions: 1) In the image (**Figure 3A**), the crop rows present a trend of being smaller at the top and more prominent at the bottom and leaning towards the center position, while the crop rows at the center position have a smaller or almost vertical inclination. 2) Vertically projecting the image exhibits a specific Gaussian distribution pattern. The maximum column pixel value position is typically located near the central crop row. The central 35% of the image is set as the region of interest (ROI) to obtain accurate results. The column pixel values within this region are then analyzed. The vertical line corresponding to the maximum column pixel value is considered the baseline, as shown in **Figure 3B**.

### 2.3 Optimal region detection

#### 2.3.1 Enhanced DBSCAN Clustering with KD-Tree Optimization

**Figure 4:**
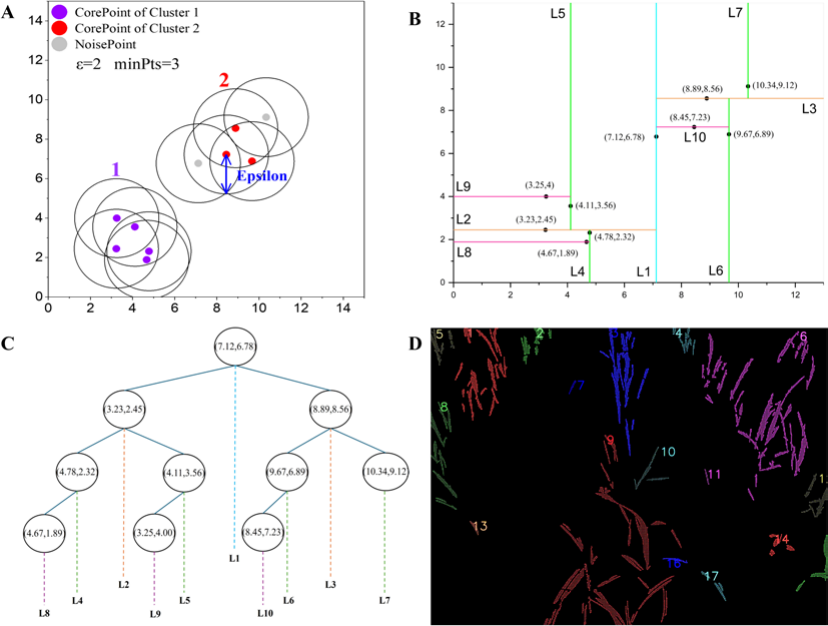
Images of clusters based on density: **(A)** Illustration of DBSCAN; **(B)** 2D space partitioning based on KDTree, each vertical and horizontal line represents a split along the x or y axis, respectively, the labels (L1, L2, etc.) indicate the hierarchical levels of the partition; **(C)** KDTree structure; **(D)** Indexes 1 to 17 indicate clusters $c_{1}\ldots c_{17}$ of that density level.

This study employed a density-based clustering method to group pixels that belong to the same crop or specific crop row, as shown in **Figures 4A, D**.

Before the implementation of clustering, it was observed that the number of pixels presented in high-resolution binary images was quite substantial, resulting in a significant utilization of computational resources and time. From the pixel reduction perspective, a sliding window method was applied to reduce unnecessary pixels, save computational time, and increase data processing speed. This method involves computing the average coordinates of all pixels within the window, as shown in Equations 12 and 13, subsequently replacing the original pixels with the resulting mean values. The sliding window moves by its step size at each iteration, covering the entire image. Through the implementation of this method, image processing is becoming more streamlined and efficient.


(12) $$\begin{array}{c} \bar{x}=\frac{1}{mn}\sum_{i=1}^{m}\sum_{j=1}^{n}x_{ij} \end{array}$$

(13) $$\begin{array}{c} \bar{y}=\frac{1}{mn}\sum_{i=1}^{m}\sum_{j=1}^{n}y_{ij} \end{array}$$

where m and n represent the width and height of the sliding window respectively, they are both set to eight in this step.

Ester et al. (1996) proposed the DBSCAN (Density-Based Spatial Clustering of Applications with Noise) algorithm. We considered two critical parameters in the algorithm, the neighborhood radius (epsilon) and the minimum number of neighbors (minPts). Based on the pre-test, the parameters were configured to 200 and 4 respectively.

The traditional DBSCAN algorithm faces computational bottlenecks, particularly when processing high-resolution images. The computational complexity of finding neighboring points increases significantly with the amount of pixel information, leading to longer processing times. To address this, the KD-Tree was introduced as a spatial indexing structure to accelerate neighbor searches in the DBSCAN algorithm, reducing the computational complexity from *O(n^2^)* to *O(n log n)*. The KD-Tree implementation, as shown in **Figures 4B, C**, was further enhanced with OpenMP parallelization to reduce processing time for high resolution images. By leveraging OpenMP, the neighbor search process was divided into smaller tasks distributed across multiple threads, significantly improving computational efficiency. The specific steps for the enhanced DBSCAN process are described as follows:

Step 1: Build a KD-tree using the dataset to accelerate neighbor searches. For each data point, find all points within its epsilon-neighborhood using the KD-tree and cache these neighbors for quick access. Perform parallel neighbor searches using OpenMP.
Step 2: Initialize all data points as unclassified by setting their cluster IDs to -1.
Step 3: For each unclassified data point, perform the following:
  - Retrieve its cached epsilon-neighborhood from the KD-tree.
  - **If** the number of neighbors (including the point itself) is less than the user-defined minimum (minPts), **then**:
    - Mark the data point as noise (remain unclassified).
  - **Else if** the number of neighbors is greater than or equal to minPts, **then**:
    - Assign the data point to a new cluster by setting its cluster ID to a new unique value.
    - Initialize a queue and add the current data point to it to begin expanding the cluster.
Step 4: While the queue is not empty, perform the following to expand the cluster:
  - Dequeue a data point from the queue.
  - Retrieve its cached epsilon-neighborhood.
  - **If** the number of neighbors is greater than 1 (meaning the point has at least one neighbor besides itself), **then**:
    - For each neighbor in the epsilon-neighborhood:
      1. **If** the neighbor is unclassified (cluster ID is -1), **then**:
        - Assign it to the current cluster by setting its cluster ID to the current cluster ID.
        - Enqueue the neighbor to process its neighbors in subsequent iterations.
Step 5: Repeat **Steps 3** and **4** for all data points until all points are classified either into clusters or marked as noise.

The corresponding algorithm for the process mentioned above is presented in **Algorithm 2**.

Algorithm 2 **The algorithm of DBSCAN using KD-tree acceleration.**

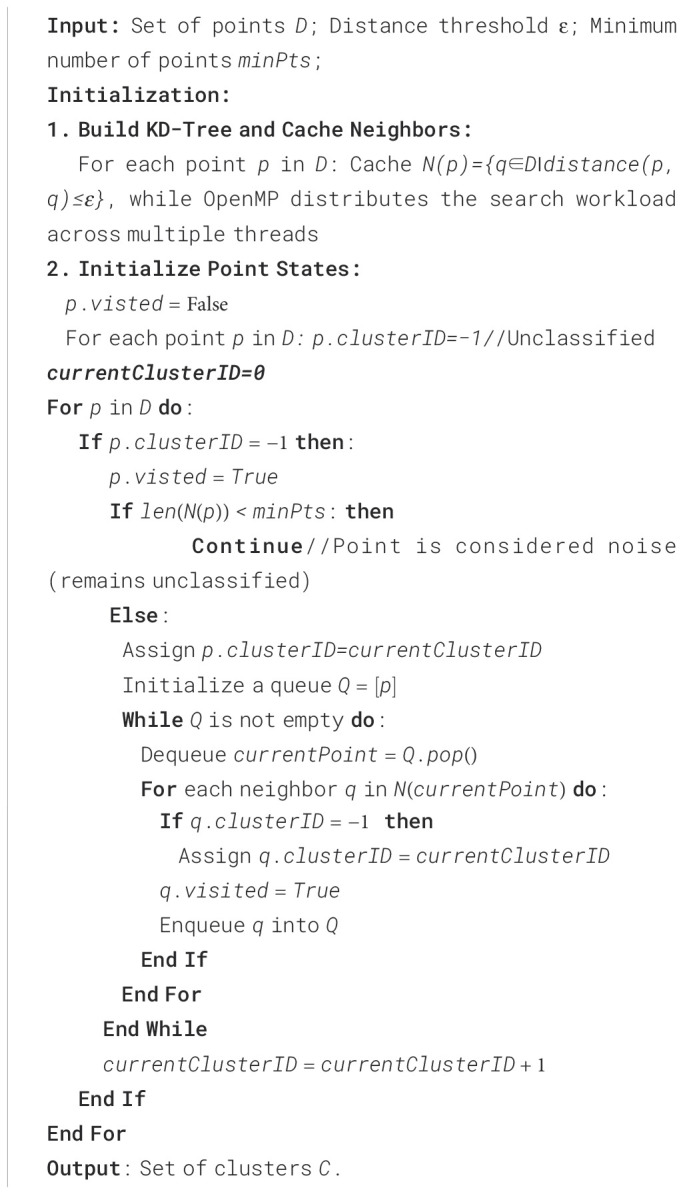

#### 2.3.2 Centerline selection by distance to the baseline

**Figure 5:**
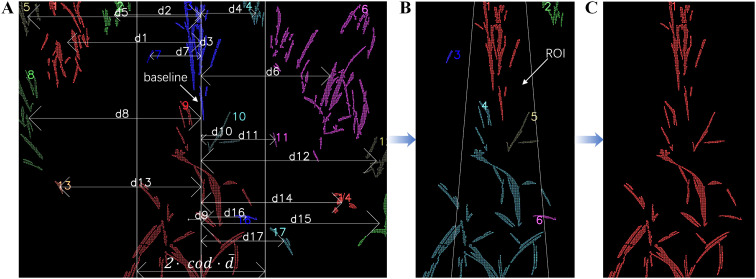
Selection of median row: **(A)** Compare $d_{1}\ldots d_{17}$ with $cod\cdot \bar{d}$ ; **(B)** Detect the presence of clusters to verify if there is at least one point within the region of interest; **(C)** Obtain the centerline.

The plantation row that is required to fit the navigation line is considered the centerline in **Figure 3A**. By conducting a comparative analysis of the distances of clusters to the baseline depicted in **Figure 3B** and subsequently delineating a specific ROI, a more precise estimation of the centerline can be obtained, as shown in **Figure 5C**.

The comparative analysis compares the distance from each cluster centroid to the baseline with the mean distance of all cluster centroids to the baseline, as shown in Equation 14. This approach enables the extraction of potential target clusters while filtering out clusters that exhibit a minimal proportion of areas within the ROI, yet the centroid is distant from this region, as illustrated in **Figure 5A**.


(14) $$\begin{array}{c} d_{i}\leq cod\cdot \bar{d} \end{array}$$

where $d_{i}$ represents an absolute distance from i - th cluster centroid of its x-coordinate $c_{i,\;\;x}$ to the baseline, $\bar{d}$ is calculated as the mean of all centroid distances and the coefficient of distance (cod) will start with an initial value of 0.65 and increase by 0.05 in each iteration until at least a cluster that satisfies Equation 14 is found.

However, not all clusters that satisfy Equation 14 are necessarily relevant to the required fitting clusters. These clusters may encompass redundant significant connected components and certain biased positional crops. To address this issue, an isosceles trapezoidal ROI with the baseline as its median was employed. **Figure 5B** illustrates the ROI, which consists of two oblique lines, each spanning from the top to the bottom, with a length equivalent to 0.07 times the image width. Furthermore, the slopes of these two lines have an absolute value of 1/12. The cross-product operation, as shown in Equation 15, was applied to assess the position of the cluster relative to the oblique lines. Equation 16 determines whether the cluster is within the ROI. The clusters contributing to a non-zero value in Equation 16 are regarded as the desired median row, as illustrated in **Figure 5C**.


(15) $$\begin{array}{c} f\left(a,b,c\right)=\{\begin{array}{c} -1\;\;\;\;\;\;\text{if}\left(b_{x}-a_{x}\right)\left(c_{y}-a_{y}\right)-\left(b_{y}-a_{y}\right)\left(c_{x}-a_{x}\right)>0\\ 1\;\;\;\;\;\;\;\;\;\text{if}\left(b_{x}-a_{x}\right)\left(c_{y}-a_{y}\right)-\left(b_{y}-a_{y}\right)\left(c_{x}-a_{x}\right)<0 \end{array} \end{array}$$

where a and b represent two points on the oblique line, c represents the point which the relative position is to be determined. The number of points within the two oblique lines is given by:


(16) $$\begin{array}{c} \sum_{i=1}^{n}I\left(f\left(l_{p,1},l_{p,2},p_{i}\right)=1\wedge f\left(r_{p,1},r_{p,2},p_{i}\right)=-1\right) \end{array}$$

where $l_{p,1}$ and $l_{p,2}$ determine the left oblique line, $r_{p,1}$ and $r_{p,2}$ determine the right oblique line, $p_{i}=\left(x_{i},y_{i}\right)\in c_{i}$ , and I(x) is the indicator function, when x is true, I(x)=1 ; when x is false, I(x)=0 .

#### 2.3.3 Sigmoid thresholding method based on NZPR

**Figure 6:**
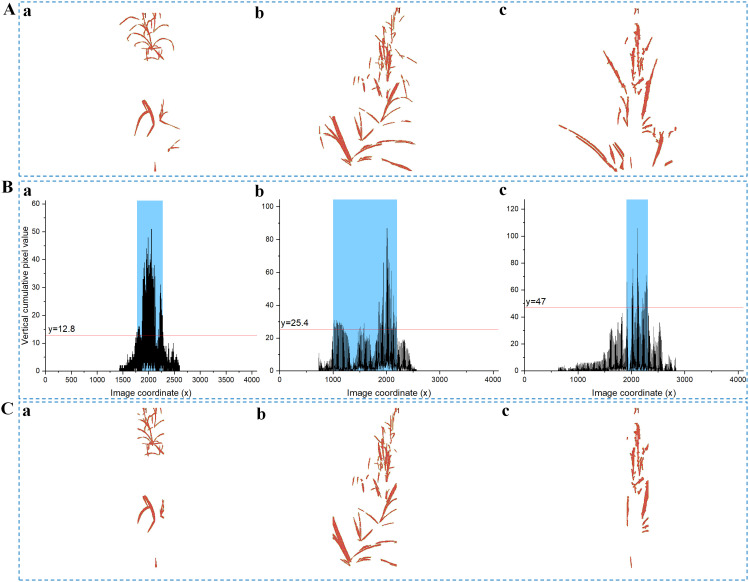
Sigmoid thresholding process. **(A)** Median rows at different growth stages: **(Aa)** NZPR=1.84%, t =0.95; (Ab) NZPR=8.96%, t =0.91; (Ac) NZPR=22.32%, t =0.76; **(B)** Vertical projection curves of centerlines: the horizontal line is applied in (Ba), (Bb) and (Bc) to determine the blue area which corresponds to the final fitting area; **(C)** Centerlines after segmentation, the larger the NZPR, the more likely it is to segment a smaller fitting area.

In crop analysis, accurately selecting a segmentation representing the crop row is essential for fitting a navigation line. Despite the series mentioned above of processing steps, it was observed that the centerline depicted in **Figure 6A** may still contain discrete branch portions, which can lead to a decrease in fitting accuracy. To mitigate this issue, a novel thresholding technique is proposed in this study, which utilizes a sigmoid function based on NZPR to segment centerline images.

The threshold for segmenting is determined by Equation 17, which maps different NZPR values to ranges from 0 to 1, normalizing pixel values in the image. Images with higher NZPR values imply later growth stages with denser median crop rows, particularly in the central stem region. Consequently, there is an urgent need for segmentation in such areas, which corresponds to a lower threshold. The results of several tests conducted on multiple crop images at various growth stages showed that k takes a value of (-8.67) and x takes a value of 0.354, resulting in relatively favorable outcomes concerning image segmentation.


(17) $$\begin{array}{c} \text{t}=\frac{1}{1+e^{-k\left(\text{NZPR}-x\right)}} \end{array}$$

As illustrated in **Figure 3B**, the vertical projection method will be reused on centerline images to carry out the segmentation process mentioned above. Specifically, a horizontal line will be generated to intersect with the vertical projection curve, and the region enclosed by the intersection points is considered the final fitting area. The horizontal line shown in **Figure 6B** is given by Equation 18:


(18) $$\begin{array}{c} line=\left(1.2-t\right)\cdot y_{max} \end{array}$$

where $y_{max}$ represents the maximum y-value on the vertical projection curve of the centerline image. The value 1.2 is optional, and since the origin of the coordinate system in OpenCV is in the top-left corner, the corresponding horizontal line y-value should be calculated starting from the origin.

### 2.4 The straight-line fitting based on RANSAC and the least squares method.

After the aforementioned preprocessing steps, the resulting image was nearly devoid of noise or outliers. Based on the resulting image, this study utilized the advantages of the RANSAC algorithm (Fischler and Bolles, 1981) to select a group of representative points with higher density. Subsequently, the least squares method shown in Equation 21 was applied to fit these points, avoiding the fitting line biased towards the density center of the points. The fitting effect is improved, as shown in **Supplementary Figure S2C**. Furthermore, as depicted in **Figure 6C**, it was worth noting that not all the pixels are required for navigation line fitting. The inclusion of additional pixels leads to increased calculative complexity. As a result, feature points that can capture the main characteristics of the crop should be selected before the fitting process. The aforementioned sliding window method is an effective technique to extract feature points. As shown in **Supplementary Figure S2A**, the data has been significantly simplified while effectively preserving the primary characteristics of the crop. In this case, the sliding window has a width of 16 and a height of 32.

The RANSAC is a widely used robust estimation technique that is critical in various computer vision and machine learning applications. The RANSAC algorithm can be expressed as shown in Equation 19. Two crucial parameters of RANSAC include the distance threshold and the number of iterations. The distance threshold can be chosen based on either empirical observations or experiments, and this study sets it to 0.155. The number of iterations is given by Equation 20.


(19) $$\begin{array}{c} L^{*}=\arg max_{\left(a,b\right)\in L}|\left\{p\in P|d\left(p,ax+b\right)<t\right\}| \end{array}$$

where $P={\left\{\left(x_{i},y_{i}\right)\right\}}_{i=1}^{n}$ represents the given set of points, L={(a,b)} represents the parameter set for the line to be fitted, d is the distance from the point P to the line, $L^{*}$ is the optimal parameter for the line, |·|  represents the size of the set and t represents the distance threshold.


(20) $$\begin{array}{c} k=\frac{\text{log}\left(1-CL\right)}{\text{log}\left(1-p^{n}\right)} \end{array}$$

where CL represents the probability of the correct model, $p=\frac{n_{inliers}}{n_{inliers}+n_{outliers}}$ represents the probability of the correct model and n which is set to 2 represents the minimum number of points required for each model estimation. To enhance model accuracy, CL is set to 0.99 in this study and $n_{inliers}$ takes a value of 2 in the initial iteration.


(21) $$\begin{array}{c} \left(a^{*},b^{*}\right)=\text{arg}{\text{min}}_{\left(a,b\right)\in L}\sum_{p\in I}{\left(y_{i}-ax_{i}-b\right)}^{2} \end{array}$$

where I={p∈P|d(p,ax+b)<t} represents the optimal inlier set.

### 2.5 Evaluation metrics

A set of crop images at different growth stages was selected for rigorous testing and evaluation to assess the efficacy of the aforementioned image-processing techniques. To quantitatively analyze the effectiveness of the image processing results, this study employed a manual annotation method to obtain ideal reference lines for crop row navigation in different images, and the obtained reference lines were regarded as a criterion. The reference lines were subsequently juxtaposed with the fitting lines, and the accuracy of the system was evaluated by computing the angular discrepancies between the reference lines and the fitted lines. The yaw angle between the fitting line and the reference line is calculated by the following Equation 22:


(22) $$\begin{array}{c} \cos\alpha =\frac{\vec{P_{1}}\cdot \vec{P_{2}}}{|\vec{P_{1}}||\vec{P_{2}}|}=\frac{x_{1}x_{2}+y_{1}y_{2}}{\sqrt{\left(x_{1}^{2}+y_{1}^{2}\right)\left(x_{2}^{2}+y_{2}^{2}\right)}} \end{array}$$

where $\vec{P_{1}}=\left(x_{1},y_{1}\right)$ and $\vec{P_{2}}=\left(x_{2},y_{2}\right)$ represent the vectors of two lines, respectively. To enhance the analysis of angular data, the following mathematical metrics for evaluation are employed: MEA, RMSE, and MRE, which are defined below.


(23) $$\begin{array}{c} \bar{\alpha }=\frac{\sum_{i=1}^{n}\alpha_{i}}{n} \end{array}$$

(24) $$\begin{array}{c} RMSE=\sqrt{\frac{1}{n}\sum_{i=1}^{n}\alpha_{i}^{2}} \end{array}$$

(25) $$\begin{array}{c} MRE=\frac{1}{n}\sum_{i=1}^{n}|\frac{\alpha_{i_{fitting}}-\alpha_{i_{annotation}}}{\alpha_{i_{annotation}}}|\times 100\% \end{array}$$

where $\bar{\alpha }$ and $\alpha_{i}$ represent the MEA and the yaw angle between two lines of i−th image, respectively, $\alpha_{i_{fitting}}$ and $\alpha_{i_{annotation}}$ represent, respectively, the angles between the fitting line and the manually annotated line with the horizontal line at the bottom of the i−th image. n is the total number of image samples.

The smaller the MEA is, the closer the fitting result is to the reference line. RMSE takes into account the size and distribution of the errors. MRE offers insights into the smaller the angle error relative to the reference line.

### 2.6 Interface design and development of field navigation

To facilitate effective user interaction and visual feedback, a human-computer interaction interface was designed and developed in this section, as illustrated in **Supplementary Figure S3**. This interaction interface contains resolution selection, processing, processing time and result display. This interface serves as a critical bridge between the algorithm and its real-world applications. Once finishing the resolution selection and processing, the result can be shown in the interaction interface.

## 3 Experimental results

### 3.1 Evaluation of cross-growth-stage sugarcane seedling images

**Figure 7:**
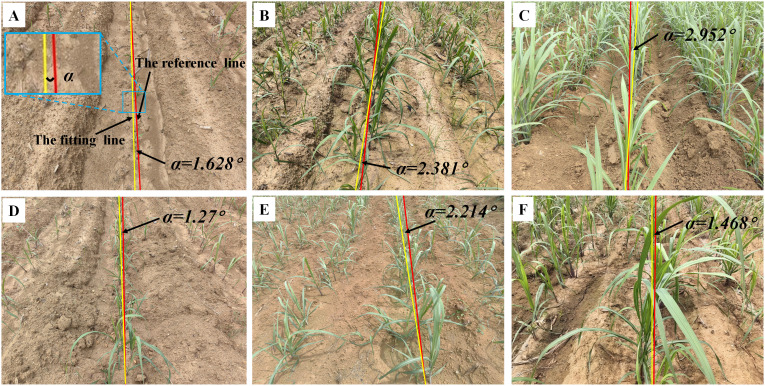
Navigation line extraction results of sugarcane seedlings with different growth stages. **(A)** Code 12; **(D)** Code 14; **(B, E)** Code 16; **(C, F)** Code 19; **(A–F)** illustrate the yaw angles of sugarcane at different growth stages.

This section involves carefully curating a subset of sugarcane seedling images from ‘Code 12’ to ‘Code 19’ to serve as a validation dataset for assessing the efficacy of the image processing techniques on sugarcane. The yaw angles between the fitting lines and the reference lines are illustrated in **Figure 7**.

**Table 1** illustrates the evaluation of three growth stages, revealing an initial upward trend followed by a subsequent decline in MEA, RMSE, and MRE. This pattern can be attributed to variations in branch distribution. Specifically, during stages of ‘Code 12-13’, sugarcane seedling foliage is small and less dispersed, resulting in minimal data fitting dispersion and relatively good symmetry. However, during stages of ‘Code 14-16’, the foliage becomes more extensive and dispersed, leading to increased data fitting dispersion and diminished symmetry. Notably, in stages of ‘Code 17-19’, the foliage is large and evenly distributed, resulting in a high degree of data fitting dispersion but maintaining good symmetry.

**Table 1:** Evaluation results of sugarcane seedling.

Growth stage	Number of samples	MEA	RMSE	MRE
Code 12-13	20	2.21°	3.35°	2.37%
Code 14-16	20	2.44°	3.86°	3.47%
Code 17-19	20	2.31°	3.61°	2.64%

### 3.2 Generalization capability verification: evaluation of seedling images in corn and rice

**Figure 8:**
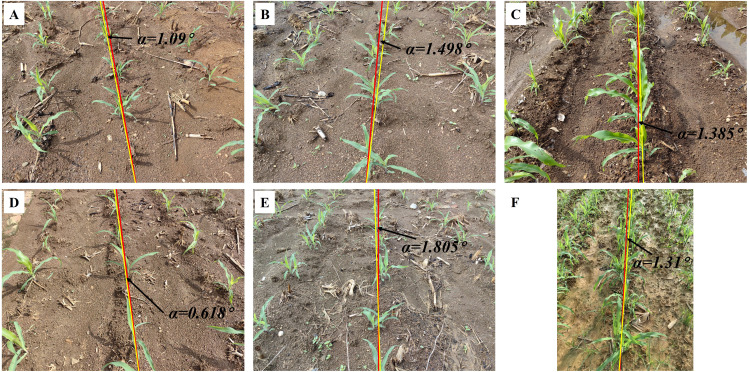
Navigation line extraction results of corn seedlings with different growth stages. **(A, B, D, E)** Code 12-15; **(C, F)** Code 16-19; **(A-F)** illustrate the yaw angles of corn at different growth stages.

**Figure 9:**
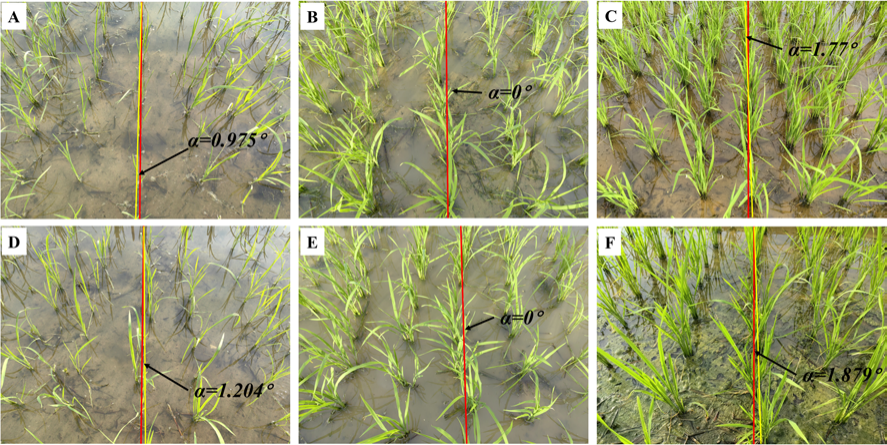
Navigation line extraction results of rice seedlings with different growth stages. **(A)** Code 12; **(D)** Code 15; **(B, E)** Code 17; **(C, F)** Code 19; **(A-F)** illustrate the yaw angles of rice at different growth stages.

This section provides a comprehensive evaluation of the image processing techniques applied to corn and rice seedling images, primarily focusing on the growth stages corresponding to ‘Code 12-15’ and ‘Code 16-19’, which aids in assessing the algorithm generality and applicability across crop image scenarios. The yaw angles between the fitting lines and the reference lines are illustrated in **Figures 8** and **9**, respectively. The evaluation of corn and rice seedlings is denoted in **Tables 2** and **3**, respectively.

**Table 2:** Evaluation results of corn seedling.

Growth stage	Number of samples	MEA	RMSE	MRE
Code 12-15	20	1.13°	1.46°	1.49%
Code 16-19	20	1.65°	2.22°	1.84%

**Table 3:** Evaluation results of rice seedlings.

Growth stage	Number of samples	MEA	RMSE	MRE
Code 12-15	20	2.96°	4.65°	3.37%
Code 16-19	20	1.679°	2.15°	1.859%

**Table 2** presents the evaluation results for two growth stages of corn seedlings. In the growth stage of ‘Code 12-15’, the algorithms exhibit enhanced performance on corn seedlings, with lower MEA, RMSE, and MRE compared to crops in the growth stage of ‘Code 16-19’. The primary factors contributing to this phenomenon are the morphological differences in corn during these two growth stages and weeds. As the growth period progresses, both the dispersion of corn leaves and the presence of weeds in the field can, to some extent, impact the accuracy of navigation line extraction.

Contrary to corn, the algorithms demonstrate better performance in the growth stage of ‘Code 16-19’ of rice compared to the growth stage of ‘Code 12-15’. The uneven dispersion of leaves in rice during the early growth stage has been well documented and attributed to the long length of the leaves. As the plant grows and more leaves are produced, the dispersion becomes more even.

### 3.3 Evaluation of ridges with seedling absence

**Figure 10:**
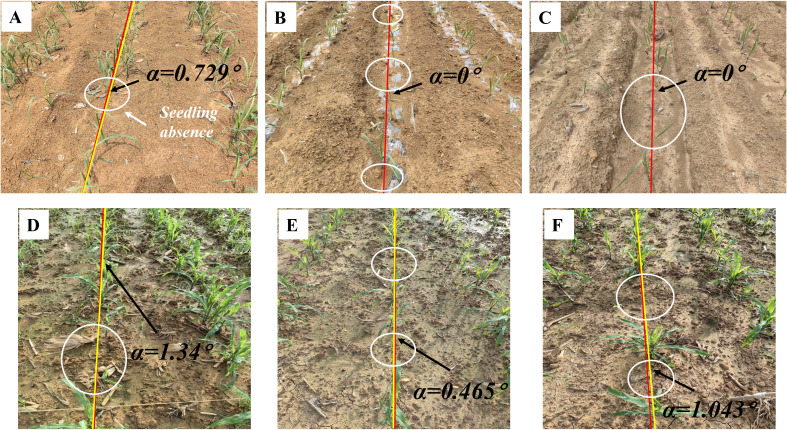
Navigation line extraction results of seedling less ridges. **(A-F)** illustrate instances of crop seedling absence within specific sections of a crop row. The white circle indicates the location of the seedling absence.

Seedling less ridges are prevalent in agricultural cultivation (see **Figure 10**), especially for multi-year crops. Such ridges result in the wastage of land, fertilizers, and other resources, ultimately impacting crop quality and yield. Achieving precise navigation in rows affected by seedling-less ridges ensures efficient execution of tasks such as replanting and fertilization application.

As shown in **Figure 10**, the navigation line can be extracted even in severe crop seedling absence in the crop rows. The MEA, RMSE, and MRE indicators in **Table 4** also demonstrate that the extracted navigation line meets high precision requirements.

**Table 4:** Evaluation results of seedling less ridges.

Environment	Number of samples	MEA	RMSE	MRE
Seedling absence	20	0.844°	1.263°	1.0448%

Based on the analysis in Section 2.2.3, the imaging method employed in this study identifies the baseline as the area with the highest pixel density along the vertical direction of the image, corresponding to the potential central crop row. By establishing the region of interest based on this baseline, the algorithm can dynamically classify crops belonging to the same row, unaffected by missing crops. The accurate extraction of the median crop row within the ROI enables the algorithm to effectively handle seedling absence. By comparing the distances between detected crop clusters and the baseline, the algorithm can robustly identify the correct row despite missing or sparse crop seedlings. This leads to improved accuracy and stability in scenarios with crop row discontinuities. These results demonstrate the centerline selection approach significantly enhances the robustness of crop row detection in challenging conditions.

### 3.4 Generalization capability verification: evaluation of diverse in-field environments

**Figure 11:**
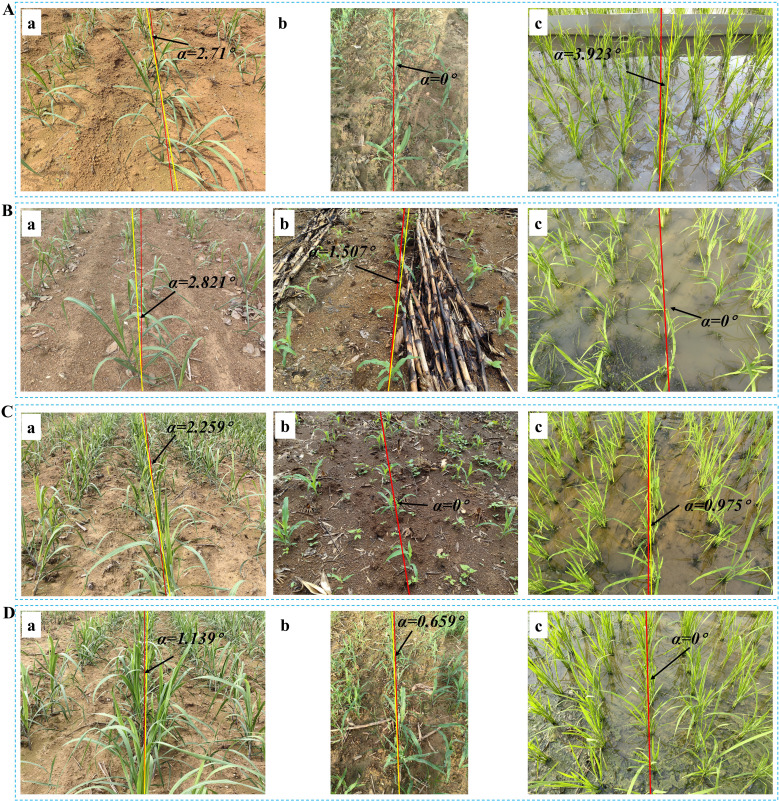
Navigation line extraction results of different crop seedlings in the complex field environment. **(A)** Variations in illumination conditions: (Aa) Intense illumination; (Ab) Shadowed environment; (Ac) Water surface glare; **(B)** Decaying branches and fallen leaves: (Ba) Dried leaves; (Bb) Large clusters of dried branches; (Bc) Fallen rice leaves; **(C)** Variations in soil backgrounds: (Ca) Yellowish soil; (Cb) Dark-colored soil; (Cc) Pale-green soil; **(D)** Chaotic vegetation: (Da) Adjacent crops interconnection and occlusion; (Db) Weed and misaligned crop; (Dc) Duckweed and green algae.

The image processing techniques presented in this study demonstrate good generalization ability for corn and rice seedlings and perform well in extracting navigation lines in complex field environments, as shown in **Figure 11**. The evaluation of diverse in-field environments is denoted in **Table 5**.

**Table 5:** Evaluation results of the complex environment.

Environment	Number of samples	MEA	RMSE	MRE
Illumination	20	0.916°	1.352°	1.008%
Branch and leave	20	1.184°	1.650°	1.330%
Soil	20	1.160°	1.716°	1.239%
Chaotic vegetation	20	0.905°	1.249°	1.008%

As shown in **Table 5**, it can be inferred that the algorithms are robust in dealing with images derived from complex in-field environments, especially for those images regarding illumination and disordered vegetation.

Compared to branch-and-leaf and soil environments, the algorithm in this study demonstrates better robustness under varying illumination conditions and in chaotic vegetation environments. This can be attributed to the algorithm primary focus on green pixels in the image, while morphological operations based on NZPR effectively eliminate redundant pixel information. However, in branch-and-leaf and soil environments, factors such as fallen leaves and the greenish appearance of soil in paddy fields introduce a certain degree of disorder to the green pixel information, resulting in a slight decrease in algorithm performance.

### 3.5 Generalization capability verification: evaluation of data augmentation for low-light and rainy conditions

Data augmentation plays a crucial role in improving the robustness and generalization capability of machine learning models, especially when addressing variations in environmental conditions. In this section, the Albumentations library was utilized to simulate real-world scenarios under low-light and rainy conditions. This evaluation aims to further validate the algorithm adaptability to special conditions and enhance its performance in practical applications.

**Figure 12:**
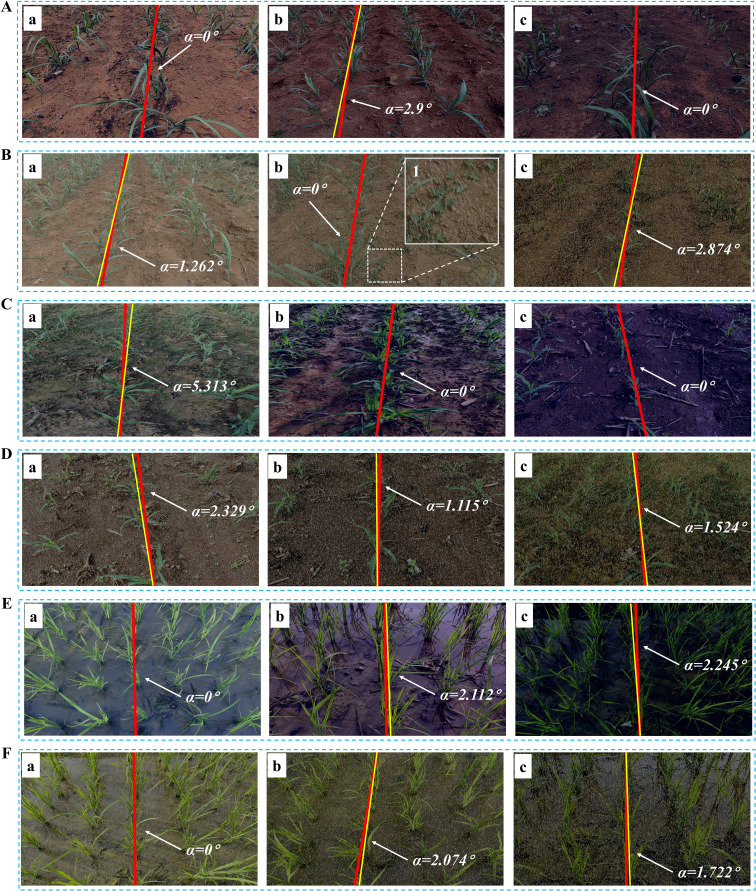
Navigation line extraction results of different crop seedlings in simulated environments. As shown in (Bb1), dim grayish raindrops are distributed across the image at a certain inclined angle. **(A)** Low light for sugarcane seedlings; **(B)** Rainy for sugarcane seedlings; **(C)** Low light for corn seedlings; **(D)** Rainy for corn seedlings; **(E)** Low light for rice seedlings; **(F)** Rainy for rice seedlings.

As shown in **Table 6**, the navigation line extraction accuracy of traditional image processing algorithms exhibits significant differences under low-light and rainy conditions. According to the data, rainy conditions have a lesser impact on navigation line extraction, with lower MAE, RMSE, and MRE values for sugarcane, corn, and rice compared to low light conditions, indicating good algorithm robustness in rainy environments. In contrast, under low light conditions, the errors increase significantly, primarily due to insufficient lighting, which restricts the extraction of green features in the images. This is particularly evident in extreme low light environments, as illustrated in **Figures 12A**c, **C**c, **E**c, where green features are substantially suppressed. Although rainy environments also involve some degree of low light, the overall lighting is typically better than in pure low-light conditions. Among the crops, corn shows the lowest error across both conditions, while rice exhibits relatively higher errors, especially in terms of RMSE and MRE, highlighting the challenges faced by the algorithm in these scenarios.

**Table 6:** Evaluation results of simulated environments.

Crop	Environment	Number of samples	MEA	RMSE	MRE
Sugarcane	Low light	20	1.723°	3.136°	1.400%
	Rainy	20	1.030°	2.105°	1.310%
Corn	Low light	20	1.478°	2.729°	1.810%
	Rainy	20	1.022°	1.813°	1.180%
Rice	Low light	20	1.698°	3.214°	1.990%
	Rainy	20	1.510°	3.042°	1.700%

### 3.6 Processing time analysis: pre- and post-optimization

**Figure 13:**
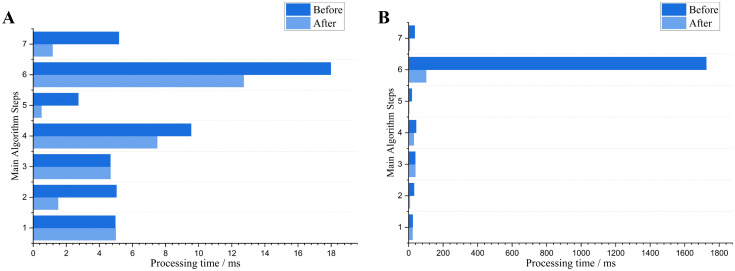
Comparison of average processing times before and after optimizations for different main algorithm modules (processing time exceeding 1 ms). The numbers 1-7 correspond to the following algorithm modules: ExG, OTSU, NZPR-Morphological Operation, Eight-Connectivity, first sliding window, KD-Tree accelerated DBSCAN, second sliding window. **(A)** Resolution of 640×480; **(B)** Resolution of 1920×1080.

In high-resolution image processing, the increase in the number of pixels necessitates the processing of a larger number of data points, which significantly augments the computational load and processing time. To address this challenge, code and algorithm optimizations were implemented. Ten images of three crop seedlings at different growth stages were used to evaluate the average time consumption of various modules in the proposed algorithm. As shown in **Figure 13**, the processing time for a late-growth stage crop image with a resolution of 1920×1080 is largely dominated by the DBSCAN clustering algorithm. This is attributed to the high complexity of the neighbor search process, where processing time escalates markedly with increasing resolution. Based on **Figure 13**, it is evident that the processing time of the optimized DBSCAN algorithm has been significantly reduced. At a resolution of 640×480, the processing time was nearly 30%. Meanwhile, at a resolution of 1920×1080, the processing time was reduced by almost 94%. However, it is worth noting that the processing time of the optimized DBSCAN algorithm still accounts for the majority of the total processing time.

**Figure 14:**
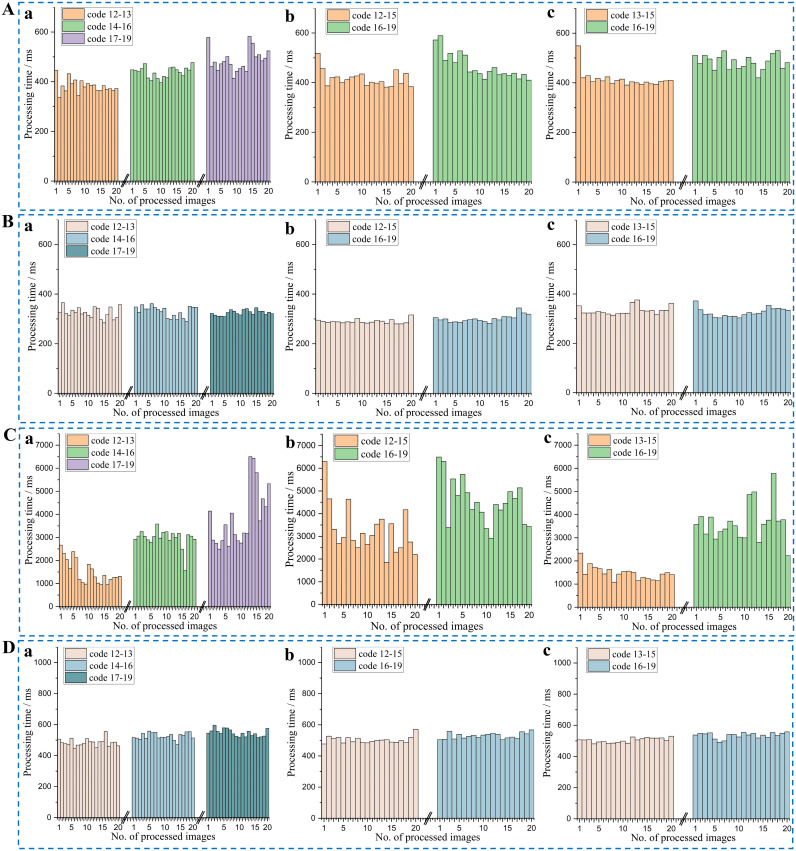
Processing times of crop images at the two resolutions. **(A)** Resolution of 640×480 before optimization; **(B)** Resolution of 640×480 after optimization; **(C)** Resolution of 1920×1080 before optimization; **(D)** Resolution of 1920×1080 after optimization; (a) Sugarcane; (b) Rice; (c) Corn.

To address the issue of DBSCAN, this study has implemented a KD-Tree to accelerate the neighbor search and utilized OpenMP for optimizing parallelization. Crop images with two resolutions (640×480 and 1920×1080) were utilized to test the processing time of the algorithm proposed in this paper, with 20 images of crops under different environmental conditions selected for each growth stage, as shown in **Figure 14**. The average processing times for the crops at these two resolutions are presented in **Table 7**.

**Table 7:** Evaluation results of the average processing time.

Resolution	Average processing time of crop seedling images/ms			
	Optimization	Sugarcane	Rice	Corn
640×480	Before	435.93	441.52	449.05
	After	326.60	295.12	329.05
1920×1080	Before	2784.10	3904.50	2502.74
	After	518.20	516.20	519.90

It is observed that processing times for crops in the early growth stages (as exemplified by instance No. 1 in **Figure 14A**b and No. 20 in **Figure 14B**b) could be comparable to or exceed those recorded in later stages (as exemplified by instance No. 15 in **Figures 14A**b, **B**b). This discrepancy is likely attributable to varying field conditions; for instance, images from earlier growth stages may contain substantial areas of weeds.

A comparison between **Figures 14A, B**, as well as **Figures 14C, D**, reveals a significant reduction in processing time after optimization, with the improvement being particularly pronounced in high-resolution image processing. Although there is some variation in processing times across different crop images, the optimized algorithm exhibits much greater stability, indicating that the optimization not only reduces processing time but also enhances the stability of performance. However, even after optimization, the processing time for high-resolution images remains significantly longer than that for low-resolution images, highlighting the continued computational challenges posed by high-resolution data.

As shown in **Table 7**, the evaluation suggests that a resolution of 640×480 offers an optimal balance for practical applications. Comparing the average processing times before and after optimization, for images with a resolution of 640×480, the average processing time decreased by approximately 25% to 30% after optimization. The optimization effect is even more pronounced for images with a resolution of 1920×1080, where the processing time was reduced by approximately 80% to 86%. The processing time was significantly reduced across all resolutions and crop types, indicating that the optimization methods employed, such as DBSCAN accelerated using KD-Tree and OpenMP parallelization, are effective in practical applications. As a result, the selection of resolution is decided by personalized needs.

## 4 Discussion

The proposed image-processing techniques exhibit effective performance in navigation line extraction for sugarcane seedling plantation rows, accompanied by a desired generalization capability when applied to different crops and diverse, complex in-field environments. This study bridges a gap that previous studies still need to address.

### 4.1 Decision process and environmental robustness of the algorithm

This study proposes a crop row navigation line extraction algorithm designed based on the principle of prioritizing green pixels. The algorithm utilizes traditional image processing techniques, starting with a preprocessing stage that enhances green features and effectively removes noise through grayscale transformation and OTSU segmentation. Morphological operations guided by the NZPR significantly reduce unnecessary pixels in the image, isolating the main stem regions of the crop rows. Noise caused by the morphological operations is further removed using an eight-connected algorithm, which also refines the stem regions. During this step, vertical projection is applied to detect curve peaks (baseline), providing a foundation for clustering the central crop row. Optimal region detection is performed using DBSCAN enhanced with KD-tree acceleration and parallelization techniques, enabling efficient pixel clustering while reducing computational time. The baseline detection process helps identify potential target crop clusters, and the trapezoidal ROI ensures the algorithm focuses on relevant areas, even in cases of crop absence, while excluding irrelevant clusters. Sigmoid thresholding further segments the target crop rows, producing the optimal region for subsequent processes. Finally, the RANSAC algorithm combined with the least squares method is employed to fit the data points. This ensures robust accuracy even in the presence of outliers, successfully extracting the navigation line. Image preprocessing is a critical step in the navigation line fitting process. During this stage, the number of extracted pixels directly impacts the algorithm performance: too few pixels may fail to adequately represent the characteristics of the crop row, while too many pixels could significantly increase the computational burden in subsequent steps. Therefore, ensuring the extracted image reflects the crop row main features is a key objective in the preprocessing stage.

**Figure 15:**
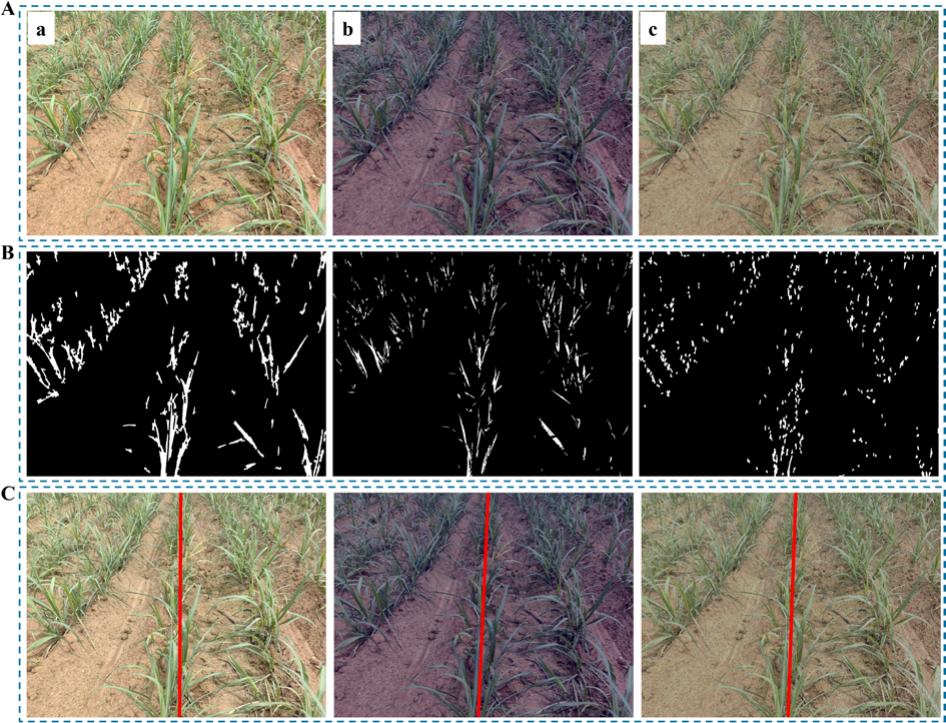
Illustration of image preprocessing under extreme environments. **(A)** Original images: (Aa) Normal; (Ab) Low light; (Ac) Rainy; **(B)** Images after preprocessing; **(C)** Results of navigation line.

To evaluate the adaptability of this step under different environmental conditions, this study specifically selected low-light and rainy conditions as experimental scenarios, given their broader and more pronounced impact on image quality compared to other factors. As shown in **Figure 15B**, the total number of pixels extracted during preprocessing is highest under normal conditions, followed by low-light conditions, and lowest under rainy conditions. This indicates that the algorithm is affected to some extent under extreme environments. However, the extracted image pixels remain effective in representing the main features of the crop rows. Moreover, the navigation lines obtained under the three different conditions are nearly identical, demonstrating the proposed algorithm robustness and adaptability in extreme environments.

### 4.2 Algorithm efficiency

In this study, 640×480 and 1920×1080 are adopted as the target resolutions for image processing. These two resolutions reflect the consideration of mainstream resolutions in current visual devices and technology trends (Lin et al., 2020; He et al., 2022). The 640×480 resolution is widely used in real-time visual processing systems since that resolution requires reduced computational power, enable efficient algorithm operation within constrained computational resources. Conversely, 1920×1080, as a representative of higher definition resolutions, provides richer details and higher image quality, suitable for applications demanding higher detail accuracy, such as precise image analysis and advanced visual recognition tasks (Yang et al., 2020). By conducting experiments at these two resolutions, the adaptability of our proposed algorithm to different application demands can be validated. When applying our proposed algorithm to perform diverse field management tasks at the seedling stage such as weeding, spraying pesticides, and root-zone soil backfill, an optimal resolution can be selected through our developed human-machine interface in response to cross growth stage of crop seedlings.

Statistical analysis reveals that the algorithm average runtime is 0.31 seconds for images with a resolution of 640×480 and 0.51 seconds for images with a resolution of 1920×1080. In practical applications, agricultural robots performing field management tasks such as weeding, spraying pesticides, fertilizer application, and root-zone soil backfill typically travel at 0.6~1.5 meters per second. This indicates that the algorithm proposed in this study addresses the demands for real-time application.

### 4.3 The algorithm adaptability to different growth stages

The excellent adaptability of the algorithm to crops at different growth stages represents a significant breakthrough in this study. The average values of the growth stages of ‘Code 12-19’ from the evaluation results of **Tables 1**–**3** show that MEA is 2.01°, RMSE is 2.95°, and MRE is 2.37%, which has demonstrated commendable performance in the case of cross-growth-stage crops. Additionally, by comparing the experimental results of different crops, it can be observed that the algorithms perform better on corn and rice seedlings, which have relatively concentrated and evenly distributed branches and leaves, than on sugarcane seedlings, which have unevenly dispersed branches and leaves.

### 4.4 The algorithm adaptability to complex field environments

An essential aspect of evaluating the performance and robustness of the proposed image processing techniques is the adaptability to complex field environments. Complex field environments, as depicted in **Figure 11**, can pose various challenges for the algorithms, such as varying illumination (**Figure 11A**), fallen leaves (**Figure 11B**a), dry branches (**Figure 11B**b), soil background (**Figure 11C**), similar colors (**Figure 11D**b) and occlusion (**Figure 11D**a), dry fields (the first and second columns in **Figure 11**) and paddy land (the third column of **Figure 11**), different crop row spacing, etc. These factors can affect the accuracy and efficiency of the algorithms in detecting and segmenting crop plants from the images. Therefore, the algorithm adaptability to different complex field environments will be discussed in this section.

On the basis of the evaluation results of environments presented in **Tables 4** and **5**, it can be inferred that MEA has an average value of 1.0018°, while RMSE has an average value of 1.446°. Moreover, MRE has an average value of 1.126%. Among the environments presented in **Table 5**, the algorithms perform the best in chaotic vegetation, indicating that they can effectively handle situations where the branches and leaves of crops overlap and occlude with each other, while also accounting for the presence of offset crops and weeds. This remarkable performance can be mainly attributed to the proposed NZPR concept and the mapping relationship between NZPR and morphological operations. It is noteworthy that the common phenomenon of seedling less ridges in agricultural production can be successfully clustered into crop rows, thanks to the centerline selection method proposed in this study.

The inter-row spacing of the crops tested in this study was 1 meter for sugarcane seedlings (**Figure 7**), 0.5 meters for corn seedlings (**Figure 8**), and 0.3 meters for rice seedlings (**Figure 9**). Upon analyzing the evaluation results of three crops, it becomes evident that the algorithms can effectively facilitate precise navigation for crops with inter-row spacing variability. As illustrated in section 2.2.3 and section 2.3.2, the utilization of vertical projection can successfully detect the baseline, which serves as the reference for centerline selection; crop clusters located in the centerline area can be determined by applying the distance threshold method, thus ensuring adaptability to inter-row spacing variability.

Arid land and paddy fields represent two major environments for crop cultivation, bearing considerable influence on agricultural production. For arid land crops, represented by corn, the average values calculated from **Table 2** present that MEA is 1.39°, RMSE is 1.844°, and MRE is 1.665%. For paddy field crops, represented by rice, the average values calculated from **Table 3** present that MEA is 2.3195°, RMSE is 3.4°, and MRE is 2.6145%. It is easily noticeable that the algorithm exhibits better adaptability to corn seedlings than rice seedlings. Apart from the influence caused by the inherent morphological differences between these two crops, factors such as reflections and glare in the paddy field environment can also interfere with the extraction of navigation lines. However, even in the case of rice seedlings, where the performance is relatively less favorable, there is a slight improvement in terms of MEA when compared to similar studies in the same category (Fu et al., 2023; Wang A. et al., 2021).

### 4.5 The algorithm adaptability to simulate environments

In practical agricultural operations, it is inevitable to encounter extreme conditions such as low light, nighttime, and rainy weather. Therefore, simulating these extreme environments, as shown in **Figure 12**, through image augmentation is highly beneficial for further validating the generalization capability of the proposed algorithm. According to the results in **Table 6**, the average values of MEA, RMSE, and MRE for the three crops under low light and rainy conditions are 1.41°, 2.67°, and 1.56%, respectively. When compared with the average values obtained under various real-world conditions in **Tables 4** and **5** (MEA: 1.0018°, RMSE: 1.446°, MRE: 1.126%), it is evident that low-light and rainy conditions present greater challenges to the robustness and generalization capability of the proposed algorithm. This discrepancy can be attributed to the fact that lighting and rain affect the image globally, leading to diminished visibility of green features under such conditions. Since the algorithm proposed in this paper heavily relies on green information for subsequent processing, the decrease of these features in low-light and rainy environments directly impacts its performance. This highlights that future research could explore the use of sensors to acquire multidimensional data of crops, addressing the limitations of relying solely on image-based information.

### 4.6 Comparative analysis between the proposed pixel-wise method and the deep learning-based method in navigation line extraction

#### 4.6.1 Navigation line extraction combining deep learning method and image processing method

**Figure 16:**
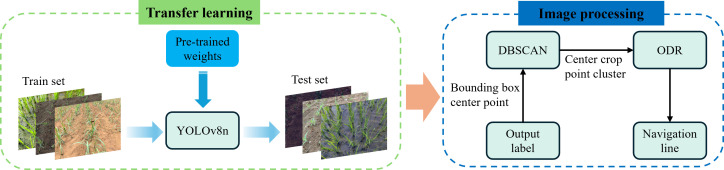
The workflow for navigation line extraction combining transfer learning and image processing methods (named as CTLIP).

On the basis of our constrained image dataset, to accelerate the training process and improve the model accuracy and generalization, we use transfer learning to obtain a model that can identify rice, sugarcane and corn plants. A YOLOv8n pre-trained weights was used in the process of transfer learning. The software environment comprised Python version 3.9, PyTorch version 2.4.1, and CUDA version 12.1. The model was trained with a batch size of 2 and 300 epochs, while all other hyperparameters were kept at their default settings. In this study, our datasets consisting three types of crop seedlings under various environmental conditions were also used for training. The primary stem regions of the seedlings were annotated, aligning with the approach of the proposed traditional image processing algorithm, which extracts the main stem regions for navigation line fitting. The workflow is illustrated in **Figure 16**. The process begins with training the YOLOv8n model on the dataset. Once trained, the model processes the test set to generate bounding boxes around the crop regions. The center points of these bounding boxes are extracted and clustered using the DBSCAN algorithm to identify the primary crop row. The clustered center points are then used in an Orthogonal Distance Regression (ODR) model to fit a navigation line.

**Figure 17:**
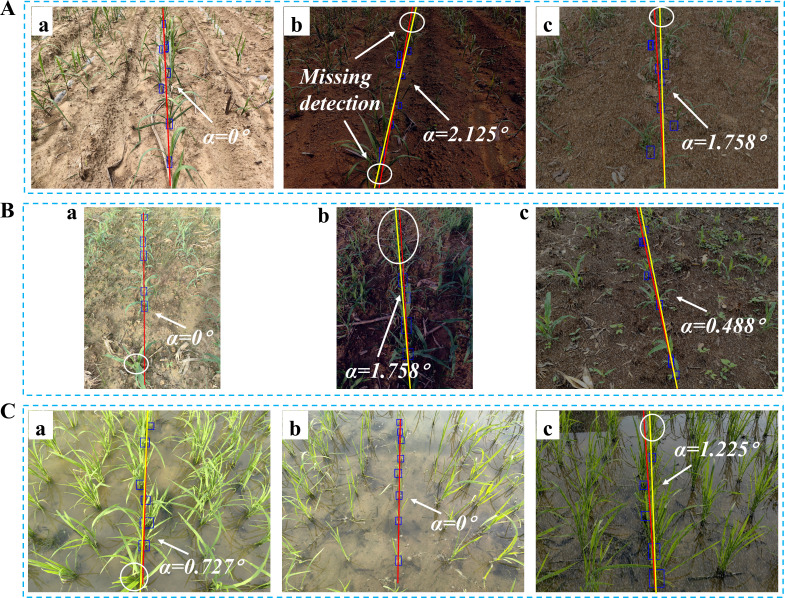
Navigation line extraction results of CTLIP. **(A)** Sugarcane seedlings; **(B)** Corn seedlings; **(C)** Rice seedlings.

**Supplementary Table S1** illustrates the evaluation results of navigation line extraction using the YOLOv8n model for three crops: sugarcane, corn, and rice. Among the crops, rice achieved the best overall performance, with the lowest MAE (0.833°), RMSE (1.225°), and MRE (0.971%), indicating that the model could effectively detect and extract navigation lines in rice fields. This performance can be attributed to the relatively distinct stem regions of rice seedlings, which are easier to identify, and the minimal occlusion between individual stems. In contrast, sugarcane and corn showed higher error values, with corn having the highest MRE (2.386%) and sugarcane having the highest RMSE (2.934°). The inferior performance of sugarcane and corn can be attributed to their severe occlusion in the later growth stages, where stem regions become nearly indistinguishable. These occluded areas significantly increase the occurrence of missed detections (shown in the white circled area of **Figure 17**), thereby affecting the accuracy of navigation line extraction. Additionally, the performance was observed to degrade under extreme low-light conditions, where green features are heavily suppressed. This leads to higher error rates and occasional missed detections. These results will serve as the basis for a comparative analysis with the algorithm proposed in this study, which will be detailed in the subsequent discussion section. The aim is to evaluate the advantages and limitations of deep learning in handling various conditions relative to the approach proposed in this study.

#### 4.6.2 Comparative analysis

To further present our study, in combination with evaluation metrics used in this study, a comparison analysis was done between our study with existing similar research and the CTLIP method, as illustrated in **Supplementary Table S2**.

As shown in **Supplementary Table S2**, the proposed navigation line extraction algorithm outperforms the traditional image processing method by Fu et al. (2023) in terms of MEA, demonstrating a notable reduction in error. Moreover, the RMSE achieved by our algorithm is 2.47°, further highlighting its accuracy across multiple crops, growth stages and diverse field environments, including sugarcane and corn, as opposed to the single crop tested in the comparative study. Additionally, the inclusion of MRE in our results presents a more comprehensive evaluation. This demonstrates the enhanced generalization and robustness of our algorithm in terms of traditional image processing. The comparison between CTLIP and the proposed algorithm indicates that both methods exhibit good performance in crop row detection. Our method achieved better results, reflecting its high precision and effective error control. This highlights the capability of traditional image processing methods to leverage prior knowledge and structured algorithms for accurate navigation line extraction in simpler, well-defined scenarios. Notably, CTLIP performed particularly well in scenarios where the main stems of crops were visible with minimal occlusion, such as in rice seedlings (lowest MEA of 0.833° in **Table 7**). Both methods outperformed the Fu et al. (2023) approach, emphasizing the adaptability across multiple crops (sugarcane, corn, and rice) and environmental conditions. The findings suggest that integrating the strengths of traditional methods and deep learning could further enhance crop row detection accuracy, particularly under diverse field conditions.

### 4.7 Error analysis of navigation line extraction

**Figure 18:**
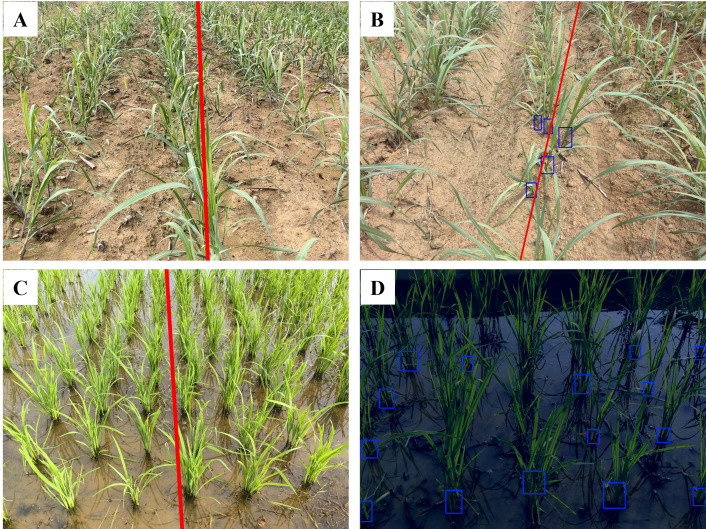
Illustrations of error samples. **(A, C)** Narrower interrow spacing; **(B, D)** Missing detection.

In this study, traditional image processing methods and deep learning-based object detection methods exhibit distinctly different challenges in navigation line extraction. For traditional image processing, a significant source of error lies in the clustering process, particularly when crop rows appear closer together at the top of the image. This issue is exacerbated in cases where the inter-row spacing is narrower, as shown in **Figure 18A, C** with rice seedlings. The clustering algorithm may mistakenly group crops from adjacent rows into the central crop row, especially in the top regions of the image, leading to inaccuracies in navigation line fitting. A practical solution to this problem is to adjust the field of view of the camera. Since real-time navigation typically does not require distant views, narrowing the field of view can reduce the apparent convergence of crop rows at the top of the image, thereby mitigating clustering errors.

In contrast, deep learning-based object detection errors primarily stem from missed detections. As illustrated in **Figures 18B, D**, missed detections result in the loss of critical information from both the top and bottom regions of the crop rows in the image. When fitting the navigation line, the algorithm can only rely on a limited number of bounding boxes, significantly impacting navigation line extraction accuracy. In extreme cases, such as when only one bounding box remains for the central crop row, it becomes impossible to perform navigation line fitting, which is a critical failure for the algorithm. The reasons for these missed detections include the inability to extract features of the main stem. This issue can arise due to severe occlusion among crops, where overlapping leaves and stems obscure the main stem from the camera view. Additionally, in low-light conditions or during inclement weather, the features of the crops become less distinct in the images. This lack of clarity hampers the deep learning model ability to accurately detect and identify the main stems, leading to increased instances of missed detections. To address this issue, future research will focus on collecting more crop datasets under varying environmental conditions and using more efficient and suitable modules in the deep learning model.

This comparative analysis highlights that, in this study, traditional methods struggle with row spacing and clustering errors, and deep learning methods are vulnerable to detection omissions, especially in adverse environmental or crop conditions. Understanding these failure modes is essential for improving the robustness of navigation line extraction in practical agricultural scenarios.

### 4.8 Advantages, limitations and prospects

#### 4.8.1 Advantages and limitations

Deep learning models have achieved impressive progress in image recognition but still have some shortcomings and challenges. One such challenge is that most existing models are trained for specific datasets and are usually unable to adapt to different scenarios, thus lacking generalization ability. Additionally, the scale and diversity of the required datasets are demanding. Furthermore, the high computational cost and latency associated with deep learning may not be conducive to some practical applications.

In extracting navigation lines, deep learning-based object detection is well-suited for scenarios where there is a clear differentiation between the growth stages, minimal crop occlusion, and easy plant identification. However, these ideal field conditions are rare in most cases. As a result, the algorithms proposed in this study fall under classical image processing techniques, characterized by their cost-effectiveness, minimal data requirements, ease of integration with edge devices, and suitability for deployment on resource-limited embedded systems or mobile devices. In addition, classical image processing techniques are more interpretable and explainable, as they rely on well-defined mathematical models and algorithms rather than black-box neural networks. This is particularly desirable for researchers, as it helps to understand the fundamental logic governing variations in phenomena, thereby enabling adequate explanation and management. Notably, deep learning-based object detection outperforms traditional image processing methods in the presence of weeds within crop rows. This advantage arises because deep learning models are capable of learning complex features and distinguishing between crops and weeds based on extensive training datasets, whereas traditional methods often rely on predefined rules and thresholds, which can be easily disrupted by the presence of similar or overlapping features such as weeds.

Overall, the presented study highlights the generalization of navigation line extraction at different crop seedling stages. Despite advancements in classical image processing techniques, there are limitations in dealing with complex image processing tasks such as complex seedling interaction between adjacent planting rows, intense weeds between plantations.

#### 4.8.2 Prospects

In this study, a pixel-wise navigation line extraction method of cross-growth-stage seedlings was verified, showing a promising solution for in-depth intelligent agricultural machinery application, especially for the field management task practices such as stumping, weeding, spraying pesticides, and root-zone soil backfill. For future studies, the proposed navigation line extraction method can be integrated into agricultural machinery, make it possible to change the current field management task practices from mechanization to intelligence, solving the problem of population ageing and labor costs.

## 5 Conclusion

From the results presented in this study, the following conclusions could be drawn:

1. The present study successfully achieved near-noiseless binary images through pre-processing. It identified appropriate fitting regions by detecting crop rows. Furthermore, it obtained precise navigation lines through linear fitting, regardless of growth stages, diverse field environments, or ridges of seedling absence.
2. The performance of the proposed algorithms was comprehensively evaluated by three indicators: MEA, RMSE, and MRE. For different crops under different growth stages or environmental conditions, MEA and RMSE are basically in the range of 1° to 4°, while MRE is in the range of 1% to 4%, which indicates that the navigation line and the reference line were closely aligned. The distribution of errors between them was found to be relatively uniform. For the 640×480 resolution, the algorithm average processing time is 310 ms, demonstrating sufficient real-time performance to meet the navigation and operational requirements of agricultural robots. For the 1920×1080 resolution, the average processing time is 510 ms, which, despite being longer, is still adequate for practical applications, ensuring that the algorithm maintains real-time functionality in field management tasks.
3. The mathematical relationship between opening operation and NZPR can create clear and distinct boundaries between crop rows in different growth stages. This enables the connected component filter to significantly suppress irrelevant information in the image. Moreover, the fitting region that best represents the central part of the crop can be obtained by applying the NZPR adaptive sigmoid function to segment the vertical projection curves of different centerlines. This ensures the accuracy of the centerline fitting.

In conclusion, this study proposed an image processing algorithm for navigation line extraction of cross-growth-stage seedlings in sugarcane, corn and rice. Its efficiency and generalization capabilities were verified in complex field environments.

## Data Availability

Publicly available datasets were analyzed in this study. This data can be found here: https://github.com/Mr-lxd/fieldNavigationProject, https://doi.org/10.6084/m9.figshare.24188097.v5.

## Supplementary material

Supplementary Figure 1 Image graying process by applying ExG. **(A)** Original image; **(B)** Grayscale image.

Supplementary Figure 2 Navigation line fitting. **(A)** Crop pixels after applying the sliding window method; **(B)** The navigation line is obtained after applying RANSAC and the least squares method.

Supplementary Figure 3 Interaction interface for field navigation at crop seedling stage.
