# Supplementary figures and images for: Pixel-wise navigation line extraction of cross-growth-stage seedlings in complex sugarcane fields and extension to corn and rice
Source: Front Plant Sci. 2025 Jan 30;15:1499896. doi: 10.3389/fpls.2024.1499896 (PMC11823478; doi:10.3389/fpls.2024.1499896)

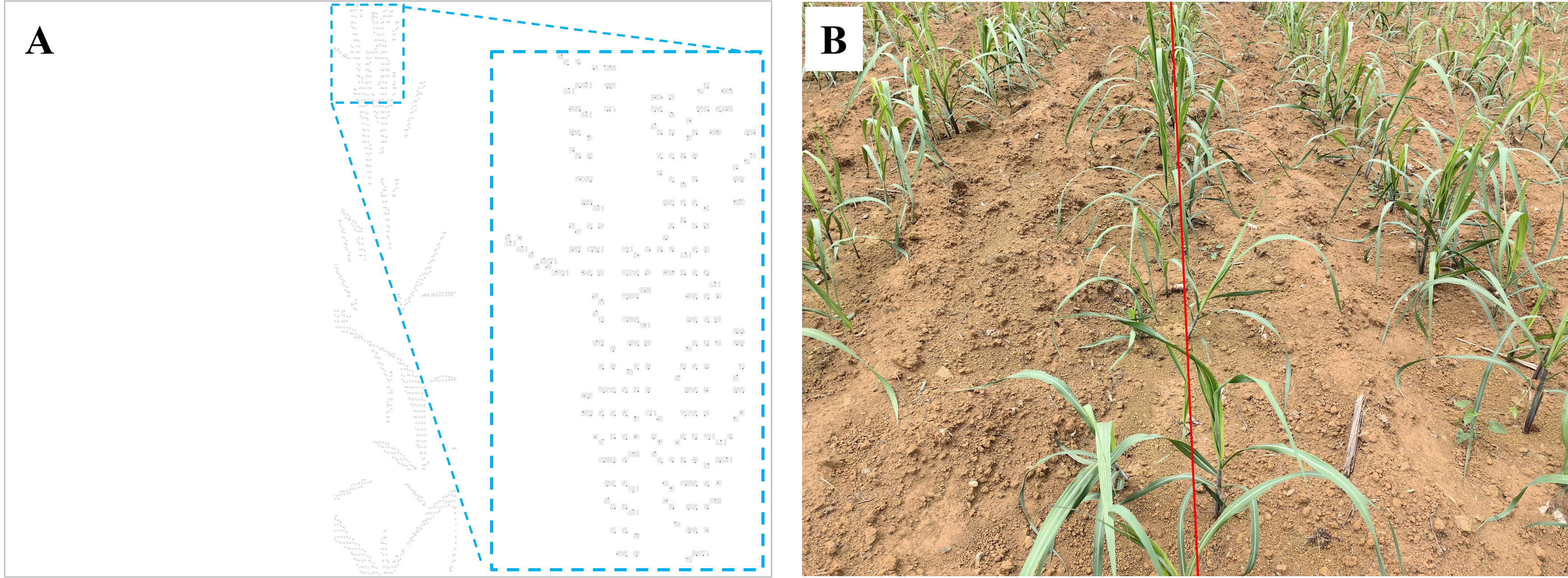

Supplement: Supplementary Figure 1 — Image graying process by applying ExG. (A) Original image; (B) Grayscale image. [file Image1.tif]

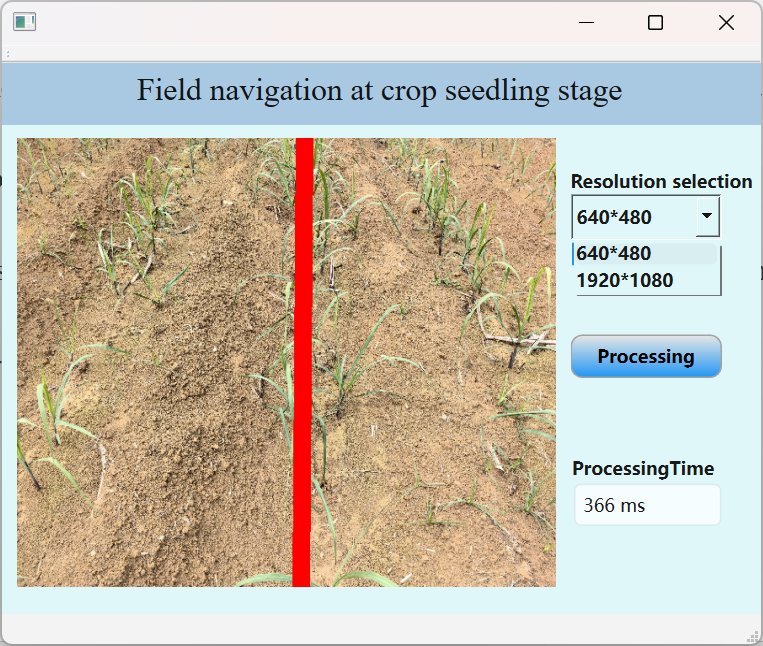

Supplement: Supplementary Figure 3 — Interaction interface for field navigation at crop seedling stage. [file Image3.tif]
