# Supplementary material for: Pixel-wise navigation line extraction of cross-growth-stage seedlings in complex sugarcane fields and extension to corn and rice
Source: Front Plant Sci. 2025 Jan 30;15:1499896. doi: 10.3389/fpls.2024.1499896 (PMC11823478; doi:10.3389/fpls.2024.1499896)
Supplement: Supplementary file 4 [file Table1.docx]

**Table S1**

Evaluation results of combining transfer learning and image processing methods.

| Crop | Number of samples | MEA | RMSE | MRE |
| --- | --- | --- | --- | --- |
| Sugarcane | 20 | 2.036° | 3.647° | 2.367% |
| Corn | 20 | 2.027° | 2.934° | 2.386% |
| Rice | 20 | 0.833° | 1.225° | 0.971% |
