# Supplementary material for: Pixel-wise navigation line extraction of cross-growth-stage seedlings in complex sugarcane fields and extension to corn and rice
Source: Front Plant Sci. 2025 Jan 30;15:1499896. doi: 10.3389/fpls.2024.1499896 (PMC11823478; doi:10.3389/fpls.2024.1499896)
Supplement: Supplementary file 5 [file Table2.docx]

**Table S2**

Comparison of crop row detection.

| Algorithms | Crop | MEA | RMSE | MRE |
| --- | --- | --- | --- | --- |
| Fu et al. (2023) | Rice | 2.34° | 2.75° | × |
| CTLIP | Sugarcane, Corn, Rice | 1.63° | 2.60° | 1.908% |
| Ours | Sugarcane, Corn, Rice | 1.54° | 2.47° | 1.78% |

Note: × indicates the missing data in relevant research. The 2.75° in this table came from two growth stages including returning green and early tillering.
